# Identification of regulators of microglia lipotoxicity

**DOI:** 10.1002/alz.089632

**Published:** 2025-01-03

**Authors:** Martine Therrien, Nicolas Weider, Giulia Monti, Daniel Meyer, Trevor Atkeson, Steven McCarroll, Anna Greka, Beth Stevens

**Affiliations:** ^1^ Broad Institute of MIT and Harvard, Cambridge, MA USA; ^2^ Broad Institute, Cambridge, MA USA; ^3^ GE HealthCare, Niskayuna, NY USA; ^4^ MIT and Harvard, Broad Institute, Cambridge, MA USA

## Abstract

**Background:**

Emerging studies have identified changes in lipid processing in Alzheimer’s disease patients. However, how the various brain cell types respond to these changes is unclear. Multiple Alzheimer’s disease risk genes are expressed in microglia and involved in lipid sensing and processing.

**Method:**

In this project, we assessed unbiasedly how various fatty acids impact microglia states and functions. Using the iPSC‐derived microglia platform, our lab has recently developed (Dolan*, Therrien* et al. Nature Immunology 2023), we characterized the impact of 48 different fatty acids on human microglia.

**Result:**

We identified 5 distinct transcriptional signatures produced in response to various lipids, including a toxic lipotoxicity signature independent of TREM2 signaling. Using microglia villages, we assessed the toxicity across 25 iPSC donors and identified genetic modifiers of microglia lipotoxicity.

**Conclusion:**

Together, our data identifies genetic regulators of microglia states and presents new insight into understanding how to protect synapses in Alzheimer’s that could lead to new therapies and biomarkers for early diagnoses.

